# Local *De Novo* Assembly of RAD Paired-End Contigs Using Short Sequencing Reads

**DOI:** 10.1371/journal.pone.0018561

**Published:** 2011-04-13

**Authors:** Paul D. Etter, Jessica L. Preston, Susan Bassham, William A. Cresko, Eric A. Johnson

**Affiliations:** 1 Institute of Molecular Biology, University of Oregon, Eugene, Oregon, United States of America; 2 Center for Ecology and Evolutionary Biology, University of Oregon, Eugene, Oregon, United States of America; University of Cambridge, United Kingdom

## Abstract

Despite the power of massively parallel sequencing platforms, a drawback is the short length of the sequence reads produced. We demonstrate that short reads can be locally assembled into longer contigs using paired-end sequencing of restriction-site associated DNA (RAD-PE) fragments. We use this RAD-PE contig approach to identify single nucleotide polymorphisms (SNPs) and determine haplotype structure in threespine stickleback and to sequence *E. coli* and stickleback genomic DNA with overlapping contigs of several hundred nucleotides. We also demonstrate that adding a circularization step allows the local assembly of contigs up to 5 kilobases (kb) in length. The ease of assembly and accuracy of the individual contigs produced from each RAD site sequence suggests RAD-PE sequencing is a useful way to convert genome-wide short reads into individually-assembled sequences hundreds or thousands of nucleotides long.

## Introduction

The decreased cost and throughput increases offered by next-generation sequencing platforms create the ability to produce high coverage of a genome in a short time. However, it remains difficult to move from many millions of short reads to a high-quality assembled genome, as the short sequence read lengths and the high error rates create computational difficulties. Several algorithms have been developed to more efficiently work with short read datasets [1], [2], [3], but these approaches require costly computing resources to compare each sequence read against all others [1], [2], [3], [4].

One difficulty in assembling a genome from short reads is bridging repetitive sequences. These sequences may exist in thousands to millions of locations in a genome, and are nearly indistinguishable in the context of a short sequence read. Without a way to place each repetitive sequence in its proper genomic location, it is difficult to move beyond producing a genome sequence made of many shorter contigs. Traditional solutions to this problem have included physically breaking the genome into smaller fragments, then cloning and sequencing each fragment independently, thereby ensuring that each repetitive sequence can be localized to a small region of the genome. The complexity reduction created by physically isolating a shorter genomic fragment is laborious, but remains one of the few true solutions to the challenges of assembling a complex genome.

RAD tags are based on a different sort of complexity reduction step that samples the DNA flanking each instance of a particular restriction site in the genome [5], [6], [7], [8]. RAD tags were developed to speed discovery of SNPs and have been particularly attractive in systems lacking a reference genome. However, moving from SNPs identified by sequencing RAD tags to a high-throughput genotyping platform is difficult without a reference genome, as these platforms typically require more than 60 nucleotides of flanking genomic DNA on both sides of the SNP of interest.

A distinctive feature of RAD tags is the asymmetric nature of the DNA fragments. Each RAD tag has one end defined by the restriction enzyme recognition site, and the other end defined by random shearing. Next-generation sequencers now have the capability to carry out paired-end reads, in which the two ends of a DNA fragment are sequenced and the two end sequences are known to belong to the same fragment. Paired-end sequencing enables RAD fragments to be used for local *de novo* assembly. A typical RAD library may contain 10,000 to 100,000 RAD sequences. The sheared-end sequences that share a common RAD-site sequence are all derived from the same small region near the RAD site. This small set of sheared-end sequences can be assembled into a larger contig. Instead of a single, computationally intense assembly using the many sequence reads from the entire genome, RAD paired-end contig assembly is performed using only a small portion of the data at a time. Because the sequence reads come from a small region, the difficulties of finding significant sequence overlap and dealing with sequence errors become simpler. To demonstrate the power of this approach, we have created RAD-PE contigs in threespine stickleback and carried out SNP discovery between two individuals. We have also created RAD-PE contigs after a partial digest with a restriction enzyme that cuts at high frequency to generate overlapping contigs in stickleback and *E. coli*. Finally, we have extended the length of the assembled contigs by including a circularization step to the library production protocol that samples a larger region near the RAD site and allows the local assembly of contigs over 5 kb in length. These methods break a genome into much smaller chunks by converting genome-wide short sequence reads into much longer high-quality contigs that are individually assembled from a small fraction of the data at a time.

## Results

### RAD paired-end contig library generation and SNP discovery in stickleback

**Figure 1:**
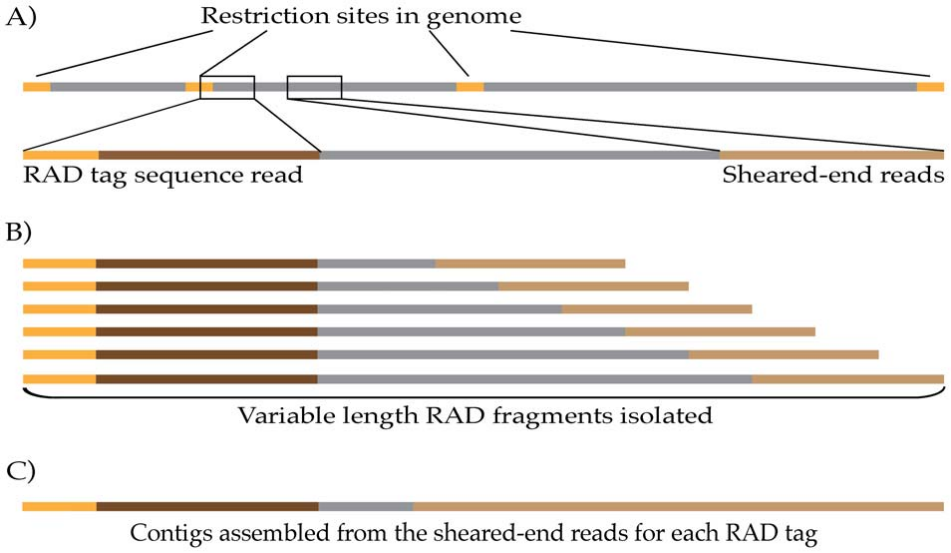
Local assembly with RAD paired-end contig libraries. (A) DNA fragments created by RAD tag library preparation have a restriction site (orange) and associated sequence (dark brown) at one end, and a random sheared-end sequence (light brown) at the other. (B) Paired-end sequencing of RAD tag libraries allows the assembly of the sheared-end sequences into contigs (C), one RAD site sequence at a time. The distance at which the random end sequence lies, and hence the length of the contigs assembled, is dictated by the size of fragments isolated during the gel extraction step in the protocol.

The DNA fragments created by RAD tag library preparation have a restriction site at one end and are randomly sheared at the other. This arrangement, when combined with Illumina paired-end sequencing, results in each instance of a restriction site sequence being sampled many times by the first reads and the genomic DNA sequence in the nearby region being randomly sampled at a lower coverage by the second reads. We hypothesized that the explicit linking of second reads that sample a genomic region with a common first read RAD sequence would allow the second reads to be assembled on a local basis, one RAD site at a time (see Figure 1).

We tested this approach by modifying the sequenced RAD tag protocol [6] in order to create paired-end compatible libraries. We altered two key aspects of the RAD protocol. First, a wider size range of fragments (300–800 bp) was isolated after shearing. The size of contigs assembled from the paired-end reads is dependent on the size range of fragments selected during library construction. Second, a longer, divergent P2 adapter that contains the reverse sequencing primer sequence was ligated to the variable end of the RAD tags before amplification, allowing the randomly sheared end of the RAD fragments to be sequenced by the second read.

For a first proof-of-concept test, we prepared barcoded *Sbf*I libraries from two threespine stickleback individuals from a phenotypically polymorphic population (High Ridge Lake, Oregon). The goals of this test were to characterize the performance of contig assembly and to determine if these RAD-PE contigs could be used for SNP discovery between samples. Barcoded samples were sequenced in a single lane of Illumina sequencing. After 2×60 bp sequencing, we obtained ∼4 million reads per sample. A custom Perl script gathered RAD sequences from the first read and kept those with at least 30 and fewer than 1000 instances. RAD sequences that are too abundant are likely to be repetitive sequences in the genome while ones that occur fewer than 30 times are unlikely to have sufficient depth of coverage along the paired-end contig to accurately call polymorphisms.

**Figure 2:**
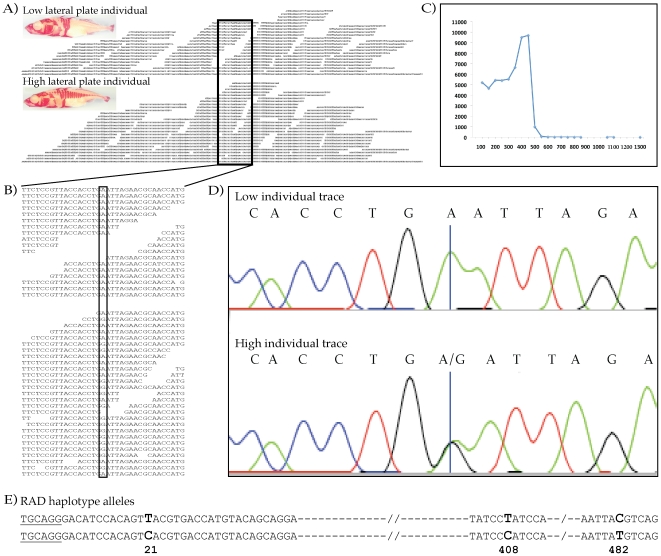
SNP identification using RAD paired-end contigs and confirmation with Sanger sequencing in stickleback. (A) An example pileup of the sheared-end reads at one RAD site from two stickleback individuals with different lateral plate phenotypes. The contig built off the reads from both individuals is shown at the bottom of each pileup, with the reads from each individual placed above the assembly. A zoom in on the region containing a SNP in one individual (bold box) is shown in (B) with the polymorphic nucleotide highlighted in the box. (C) Histogram of contig length. The N_50_ length is 407 nucleotides. (D) Validation of SNP calls by Sanger sequencing of the region surrounding the SNP identified in (B). The high plate individual was confirmed to be heterozygous at the nucleotide position identified by the analysis, while the low plate individual was confirmed homozygous and matches the consensus at that position. (E) Verified RAD haplotype alleles: an example bi-allelic RAD sequence identified in the low plate individual (shown to the left) and the relevant contig region (right). The *Sbf*I site is underlined, the SNPs confirmed by Sanger sequencing of individual amplicon clones are in bold. Nucleotide positions of polymorphisms, relative to the start of the *Sbf*I site in the reference sequence, are displayed below.

The paired-end reads associated with each RAD site were extracted and the 30–1000 sequences sent to the *de novo* assembly program Velvet [9]. Assembling the paired-end reads from each RAD site sequence one RAD sequence at a time resulted in 53,296 contigs with an N_50_ length of 407 nucleotides (Figure 2A, Files S1, S2, S3). A different Perl script took the paired-end reads of the two stickleback individuals from each RAD site and used the short-read aligner NovoAlign [10] to map the reads back to the contig and identify possible SNPs. A simple thresholding algorithm that required at least four instances of a nucleotide change was used to distinguish SNPs from sequence errors. We identified 40,441 high-quality SNPs between the two individuals in 15,152 of the contigs with an average of 2.6 SNPs per polymorphic contig (File S4).

Five contigs containing 13 polymorphisms between the two individuals were selected for validation. PCR primers were designed to target the region surrounding the polymorphisms and the products from each individual were sequenced using the Sanger method. All 13 polymorphisms (12 SNPs and a single small insertion) that were called by our analysis were verified (see Figure 2B). Both homozygous and heterozygous alleles were successfully identified.

While many of the assembled contigs are associated with a RAD site sequence present on both homologous chromosomes, polymorphisms within the RAD site sequence result in contigs specific to one of the homologous chromosomes, resolving the haplotype of the polymorphisms in the contig. We identified putative haplotypes in the contigs from one of the fish (L2-110) using the same thresholds for calling SNPs as above. Multiple individual amplicons were cloned and sequenced from each tested contig, in order to sample both haplotypes within the pool of PCR products. Sanger sequencing of the amplicons confirmed the predicted haplotypes of the three regions tested (see Materials and Methods). Figure 2E shows the haplotype sequences for one of the bi-allelic RAD tags investigated, highlighting the SNPs confirmed by Sanger sequencing. In addition, the 12 polymorphism calls (10 SNPs and 2 indels) making up the haplotype contigs in the three regions were all confirmed.

Thus, RAD-PE contigs are a robust way to generate long local assemblies at consistent regions of the genome between samples, making it a useful approach for comparative genomics, including marker and haplotype discovery.

### Partial-digest RAD paired-end libraries for whole-genome coverage in stickleback and *E. coli*

**Figure 3:**
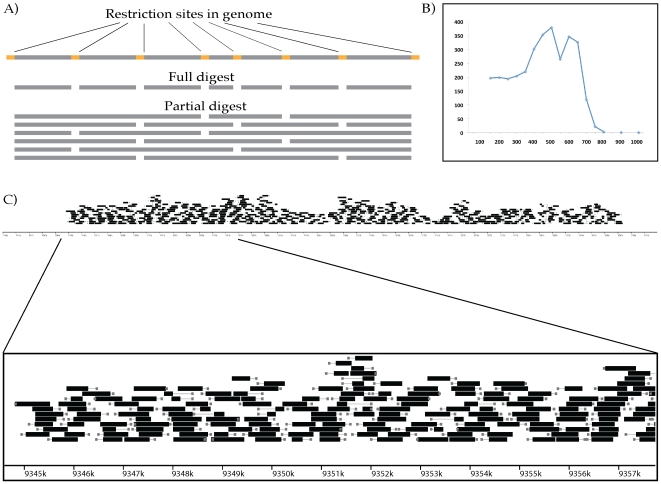
Sequencing and local assembly of overlapping contigs from stickleback fosmids using partial-digest RAD paired-end libraries. (A) Incomplete digestion of DNA with a frequently cutting restriction enzyme creates overlapping restriction fragments. Preparing RAD-PE libraries from a stickleback fosmid following partial digestion with two frequent cutters resulted in contigs up to 1000 bp long and an N_50_ individual contig length of 481 nucleotides. (B) Shows the distribution of contigs built from the two libraries. (C) Mapping the contigs (black bars) from each RAD site sequence (grey boxes) back to the stickleback reference sequence demonstrated overlapping coverage over an ∼40 kb stretch of the genome with a zoom on part of the assembly displayed below.

We modified the above protocol in order to achieve high coverage of a whole genome with RAD-PE contigs. Libraries were created by partially digesting genomic DNA with a high-frequency restriction enzyme, which produced overlapping DNA fragments several kb long that were suitable for shearing (Figure 3A). As a result, RAD cut sites are typically only a few hundred base pairs apart, but the sheared ends sample ∼500 bp regions to the left and right of each RAD site.

We first tested the performance of this partial-digest RAD-PE contig protocol by sequencing a fosmid from stickleback. After partially digesting the DNA using two restriction enzymes with different 4 bp recognition sequences, *Nla*III and *Sau3A*I, 1.0–5.0 kb DNA fragments were isolated prior to P1 ligation and shearing. Partially digested DNA samples were then carried through the RAD-PE contig protocol as described above. Because the samples were oversequenced (>3 million reads total), we removed reads that increased coverage over a 30× threshold, leaving 2 million reads for the assembly. We also tuned the Velvet parameters for each RAD site using a script that tested three different word lengths and chose the assembly with the longest total contig lengths for that site (Figure 3B, File S5). The partial-digest strategy produced overlapping contigs as predicted (see Figure 3C) with an N_50_ length of 481 nucleotides for the contigs from each RAD sequence; however, the assembled contigs mapped to two different regions of the genome, suggesting there were two fosmids present in the original prep.

**Figure 4:**
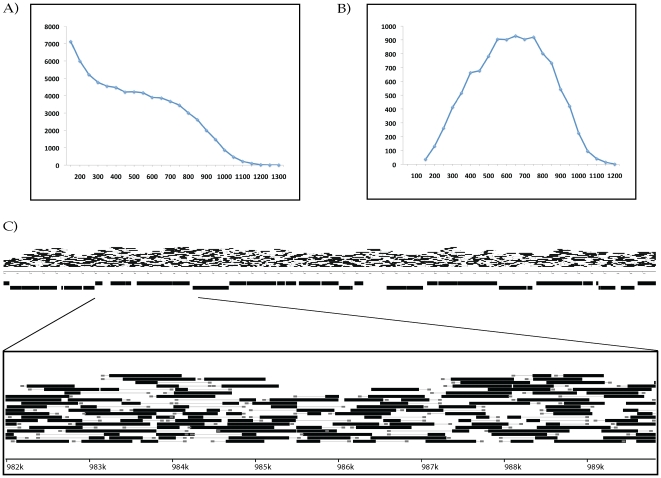
Whole genome sequencing and local assembly of overlapping contigs with partial-digest RAD paired-end libraries in *E. coli*. Partial-digest RAD-PE libraries created individually assembled contigs up to 1300 bp long with an N_50_ length of 649 nucleotides (A), or 729 nucleotides if only the longest contig was chosen from each RAD site assembly (B). (C) RAD site sequences (grey boxes) and associated contigs (black rectangles) shown for a 50 kb region of the reference genome (annotated gene regions shown as thicker black rectangles). A close-up of a smaller region is at bottom.

As a proof-of-principle for whole-genome sequencing we performed this partial-digest approach on a sequenced strain of *E. coli*. In order to maximize the possible contig length we increased the size range of DNA fragments collected to 200–1200 base pairs, but otherwise treated them the same as for the fosmid prep. From 2 million reads of an asymmetric 40×80 bp sequencing run, we identified 52,917 unique RAD sequences, sent the paired-end reads of each RAD sequence to Velvet, and assembled 70,319 contigs. The contigs assembled from each RAD sequence had an N_50_ length of 649 nucleotides (Figure 4A, Files S6, S7, S8, S9). If just the single longest contig was chosen from each RAD site assembly, the contigs had an N_50_ length of 729 nucleotides (Figure 4B).

Mapping all of the assembled contigs back to the *E. coli* reference sequence showed highly redundant coverage across the genome (see Figure 4C), with >99.9% of the genome having at least single contig coverage, and >91% of the genome having at least 5× coverage. The contigs also had a low error rate, with 54,189 of the contigs having no errors when mapped to the reference. We examined the 13,850 contigs with a single error and found that 8,642 of the errors were within 30 nucleotides of a contig end, typically a region of low coverage.

### Long-insert RAD paired-end contigs

**Figure 5:**
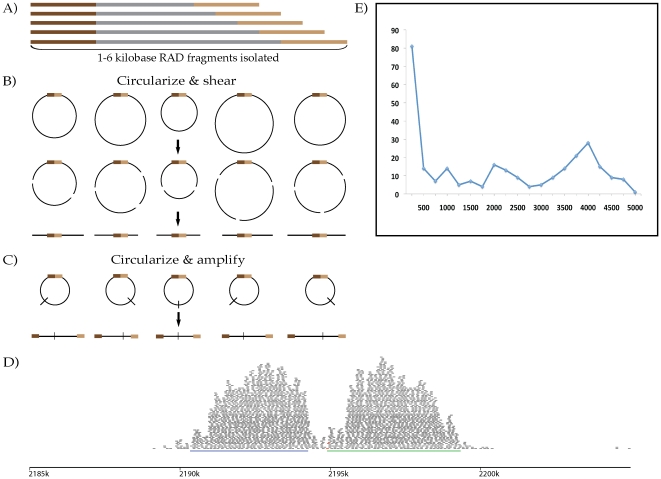
Long-insert RAD paired-end contigs. Increasing the length of RAD fragments isolated before paired-end library preparation (A) and adding two circularization steps (B, C) creates short fragments with two ends that were originally distantly separated. (D) An example of the pileup of reads (grey bars) and the resulting contigs (blue and green lines) from both sides of one *Sbf*I site (short red bars) in *E. coli* assembled from a long-insert RAD library. The individually-assembled contigs produced were up to 5 kb in length with an N_50_ length of 3,807 base pairs (E).

Illumina sequencers are not able to easily sequence DNA fragments greater than 1 kb in length, limiting the maximum possible contig length produced by the above protocols. We circumvented this limitation by adding a circularization step to bring together genomic regions up to 6 kb apart (see Figure 5). The resulting circles were sheared, re-circularized, and then linearized by PCR to create a short DNA fragment with the distantly separated genomic regions at the ends. We digested an *E. coli* genome with the restriction enzyme *Sbf*I and then made libraries that sampled 1–6 kb and 2–6 kb away from the cut sites. Contigs were locally assembled for each RAD sequence with Velvet using data from both libraries and the same processing as above to remove over-abundant reads. We took an additional computational step by first assembling the short reads with a long word length of 41, then assembling the short reads again with a shorter word length that depended on coverage and used the contigs produced by the first assembly as long reads to help bridge repeats in the sequence. We produced contigs from each RAD sequence with an N_50_ length of 3,807 bp and a maximum contig length over 5 kb (Figure 5E, File S10). Figure 5D shows an example of the pileup of reads around one *Sbf*I site and the resulting contigs assembled from each tag.

## Discussion

### SNP discovery for genotyping platforms

While RAD tags are currently used for both SNP discovery and genotyping, here we have demonstrated that paired-end RAD tag sequencing enables a local assembly step of the sheared-side short reads into high-quality long contigs. These contigs can be assembled at discrete, infrequent restriction enzyme cut sites, extending the sequence space used for SNP discovery and providing sufficient flanking sequence for high-throughput genotyping platforms such as the Illumina GoldenGate and Sequenom iPlex [11], [12], [13], [14]. RAD paired-end contigs provide several hundred nucleotides for SNP discovery and flanking sequence characterization. While these lengths can be achieved by 454 sequencing, RAD-PE contigs have the additional advantage of providing sufficient coverage depth to determine heterozygosity and reduce sequencing errors.

We demonstrated the use of RAD-PE contig libraries for SNP discovery by comparing two threespine stickleback fish DNA samples. Using less than one half of a single channel of Illumina 2×60 bp sequencing we assembled over 50,000 contigs with an N_50_ individual contig length of ∼400 nucleotides and identified more than 40,000 SNPs. Thus, many thousands of SNPs can be rapidly identified at a low cost, in a format suitable for high-throughput genotyping. Furthermore, a subset of the contigs that are associated with bi-allelic RAD site sequences have resolved haplotypes that map contig alleles to one or the other of the homologous chromosomes over the length of the contig.

### Whole genome sequencing

RAD tags are typically used to sample a portion of the genome, allowing high coverage at a desired number of loci. However, we modified the protocol to produce RAD-PE contigs that overlap over a genome by using partial-digests with a frequent cutter. Whereas whole genome shotgun sequencing requires specialized and expensive computational resources for assembly of large genomes, RAD-PE contigs can be assembled on any computer for any size genome because the genome is broken down into chunks of several hundred or several thousand base pairs that are assembled one at a time.

The many challenges of whole genome assembly are mitigated by local assembly. Short read sequences have a high error rate, so for a whole genome assembly every sequence must be searched against all others using relaxed alignment parameters. But then related regions of the genome and repeats become indistinguishable. Also, sequences must have a long region of overlap to be pieced together in whole genome assemblies, as shorter words are found throughout the genome. When RAD-PE contigs are assembled, the small region size allows for easy alignment of even high-error sequences, and short regions of overlap are sufficient to piece sequences together.

Genome assembly programs like Velvet require the user to choose parameters such as word length and expected coverage. Even the best whole genome shotgun methods create peaks and valleys of coverage across a genome and the genome itself has regions of low and high complexity. Despite this variation, during assembly a median value for each parameter is chosen and the assembler therefore is less optimal in those regions that differ from that median. Our scripts collect the reads from a particular region and attempt to optimize the assembly for that single region by removing excessive reads and adjusting the indexing word length in response to the predicted coverage, with low coverage assemblies using a short word length, allowing sparse reads to join together and high coverage assemblies using a longer word length to bridge non-unique short sequences in the region. We also routinely tried a fixed low, fixed high and this predicted optimal word length for each region and evaluated the results to choose the best assembly for further use. Velvet can be recompiled to use longer word lengths than the default maximum of 31, but this greatly increases the memory requirements for an assembly. While this is a problem for whole genome shotgun assemblies that already require hundreds of gigabytes of memory, we took this step for our assembly of the long-insert RAD-PE contigs without difficulty due to the low memory requirements of local assembly.

We showed the utility of using RAD-PE contigs for sequencing large genomic regions by performing a partial-digest RAD-PE contig approach on a fosmid from stickleback using two different high-frequency cutters. We examined the performance of partial-digest RAD-PE contigs by sequencing a strain of *E. coli*. From 2 million reads of 40×80 bp sequencing we achieved dense, overlapping coverage over the genome. Although the contigs produced can be used by current *de novo* assembly software, the optimal mix of locally-assembled contigs with other data types for whole genome assembly has yet to be tested. Also, this approach should translate well to even the largest eukaryotic genomes, however some gaps in coverage would be expected in long stretches of low complexity sequence that lacks the restriction site.

### Comparison to other methods

RAD paired-end contigs provide a low-cost method for SNP discovery in a format suitable for high-throughput genotyping platforms that require flanking sequence for primer design. It is possible to use platforms such as Roche 454 to achieve similar read lengths; however, accurate SNP discovery requires low error rates and sufficient depth of coverage to sample both chromosomes and determine heterozygosity. Although pricing of sequencing platforms rapidly change, a similar SNP discovery project using the 454 platform would have cost more than ten times as much as RAD-PE contig sequencing at the time of the project. The 8 million reads used to create greater than 50,000 contigs and find more than 40,000 SNPs between the two stickleback samples were, at that time, just one quarter of a single Illumina GAIIx lane (1/28^th^ of a run), whereas similar coverage would require at least one half of a full 454 run.

A related strategy to RAD paired-end contigs, termed subassembly, was recently described [3]. The complexity reduction step in subassembly is achieved randomly by dilution and amplification rather than restriction digestion, and subassemblies use the end sequence of the amplified fragments as an index rather than a restriction cut site sequence. As a result, subassembly does not create contigs at the same loci between samples, making the several hundred nucleotide contigs it produces useful for shotgun sequencing rather than SNP discovery.

There is justified excitement over the next generation of sequencing platforms, which promise longer read lengths and simpler informatics. The longer assembly lengths created by long-insert RAD-PE contigs match the several kilobase output projected for the next generation of high-throughput sequencers, and the local assembly step also simplifies the computational needs of a *de novo* assembly project. While the next generation of sequencers currently suffer from a high error rate, RAD-PE contigs have a low error rate due to high coverage of any particular nucleotide. Therefore, users of high count, short read length sequencers can enjoy many of the benefits of long read lengths without the considerable expense of purchasing new systems and trouble of substantially altering their workflows.

## Materials and Methods

### DNA isolation

Stickleback genomic DNA was isolated from pectoral fin clips using the DNeasy Tissue Kit (Qiagen). *E. coli* genomic DNA was acquired from the REL606 strain (provided by the Bohanan lab, UO) and from type B cells, ATCC 11303 strain (USB Corporation). Stickleback fosmids were isolated from genomic DNA using the CopyControl™ Fosmid Library Production Kit (Epicentre).

### RAD paired-end library construction for Illumina sequencing (stickleback)

1.0 µg of genomic DNA from each individual (H2 -141, L2-110) was digested for 60 min at 37°C in a 50 µl reaction volume containing 5.0 µl 10× Buffer 4 and 10 units (U) *Sbf*I-HF (New England Biolabs [NEB]). Samples were heat-inactivated for 20 min at 65°C. 4.0 µl of barcoded *Sbf*I-P1 Adapter (100 n*M*), a modified Illumina© adapter (2006 Illumina, Inc., all rights reserved; top oligo: 5′-AATGATACGGCGACCACCGAGATCTACACTCTTTCCCTACACGACGCTCTTCCGATCTxxxxxTGC*A-3′ [xxxxx = barcode (AGAGT-H2; CAGTC-L2), * = phosphorothioate bond]; bottom oligo: 5′-Phos-xxxxxAGATCGGAAGAGCGTCGTGTAGGGAAAGAGTGTAGATCTCGGTGGTCGCCGTATCAT*T-3′), was added to each sample along with 0.6 µl rATP (100 m*M*, Promega), 1.0 µl 10× NEB Buffer 4, 0.5 µl (1000 U) T4 DNA Ligase (high concentration, NEB), 3.9 µl H_2_O and incubated at room temperature (RT) for 30 min. Samples were again heat-inactivated for 20 min at 65°C, combined, and randomly sheared (Bioruptor) to an average size of 500 bp. The sheared sample was purified using a QIAquick Spin column (Qiagen) and run out on a 1.25% agarose (Sigma), 0.5× TBE gel. A smear of DNA approximately 300–800 bp was isolated with a clean razor blade and purified using the MinElute Gel Extraction Kit (Qiagen). The Quick Blunting Kit (NEB) was used to polish the ends of the DNA in a 25 µl reaction volume containing 2.5 µl 10× Blunting Buffer, 2.5 µl dNTP Mix and 1.0 µl Blunt Enzyme Mix. The sample was purified and incubated at 37°C for 30 min with 10 U Klenow Fragment (3′-5′ exo^−^, NEB) in a 50 µl reaction volume with 5.0 µl NEB Buffer 2 and 1.0 µl dATP (10 m*M*, Fermentas), to add 3′ adenine overhangs to the DNA. After another purification, 1.0 µl of Paired-End-P2 Adapter (PE-P2; 10 µ*M*), a divergent modified Illumina© adapter (2006 Illumina, Inc., all rights reserved; top oligo: 5′-Phos-GATCGGAAGAGCGGTTCAGCAGGAATGCCGAGACCGATCAGAACAA-3′, bottom oligo: 5′-CAAGCAGAAGACGGCATACGAGATCGGTCTCGGCATTCCTGCTGAACCGCTCTTCCGATC*T-3′), was ligated to the DNA fragments at RT. The sample was purified and eluted in 50 µl. 25 µl of the eluate was digested again with *Sbf*I for 30 min to remove rare genomic DNA concatemers formed from re-ligation of short fragments with two *Sbf*I restriction sites within 500 bp. The sample was purified, eluted in 50 µl and quantified using the Quant-iT™ dsDNA HS Assay Kit and Qubit™ fluorometer (Invitrogen). ∼40 ng was used as template in a 100 µl PCR amplification with 50 µl Phusion Master Mix (NEB) and 4.0 µl modified Illumina© amplification primer mix (10 µ*M*, 2006 Illumina, Inc., all rights reserved; P1-forward primer: 5′-AATGATACGGCGACCACCGA-3′, P2-reverse primer: 5′-CAAGCAGAAGACGGCATACGA-3′). Phusion PCR settings followed product guidelines (NEB) for a total of 14 cycles with an annealing temperature of 65°C. The library was cleaned through a column and gel purified, excising DNA ∼350–850 bp in size in an inverted triangle shape. PCR amplification of a wide-range of fragment sizes often results in biased representation of amplified products with an increased number of short fragments. We found this to be true in our current protocol, but reduced the effects by selecting a triangular slice during gel extraction to reduce the level of short fragment lengths from the PCR reaction. The sample was diluted to 10 n*M* and sequenced on the Paired-end module of the Genome Analyzer II following Illumina protocols for 2×60 bp reads. Sequences are available at the NCBI Short Read Archive (http://www.ncbi.nlm.nih.gov/Traces/sra; accession number SRA024496.1).

### Sequence analysis, contig assembly and SNP calling

Raw sequence reads were processed using custom Perl scripts (E.A.J., Files S11, S12, S13, S14), to optimize read number and reduce artifacts within the data. Barcodes, if present, were trimmed from the raw reads. Reads with more than 25 poor quality scores (‘K’ or worse, Illumina 1.5+ fastq) were removed. The number of reads from each RAD site was tracked, and RAD sequences above a threshold were considered repetitive and removed (1000 instances for stickleback SNP discovery [Files S11], 1500 instances for fosmid assembly [File S12], 500 instances for *E. coli* whole genome sequencing [File S13], no threshold for long-insert *E. coli* sequencing [File S9]). Single mismatch derivatives of these repetitive RAD sequences were also removed. RAD sites with a number of reads below a threshold (30 for SNP calling, 25 for fosmid and *E. coli* sequencing, and 1000 for long-insert *E. coli* sequencing) were also removed from further analysis, as the associated paired-end reads would therefore lack sufficient coverage for calling SNPs or were likely to be sequence-error created artifacts.

The paired-end reads from each passing RAD site passing the above tests were sent to the Velvet assembler (version 0.7.55) with a word length parameter that increased with increasing depth. Separate Velvet assemblies were also run with a fixed low and high word length, and the best assembly was chosen from the three trials based on the total assembled length of contigs. For the long insert assembly, the paired-end reads from each RAD site were assembled with a word length of 41. The paired-end reads were re-assembled with a predicted optimal word length based on coverage and the first assembly contigs included as long read sequences to help guide the assembly at repeats.

SNP calling was performed by aligning the sequence reads from each individual to the assembled contigs with Novoalign (version 2.07) [10]. Mismatches were filtered to include only high quality nucleotides and tracked by sample. SNPs were called using a simple thresholding.

### SNP/haplotype confirmation in stickleback

PCR primer pairs spanning contig regions, which contained putative SNPs between the individual fish, were used to amplify and sequence genomic DNA using standard Sanger sequencing protocols. The forward primer from each PCR reaction was used as the sequencing primer. Traces were analyzed using CodonCode Aligner (CodonCode Corporation). Primer pairs: contig 8041 – F 5′-CCGTATCCCAGACGCATTACAG-3′, R 5′-CGACTTGGCACTCACTAAACACAG-3′; contig 14660 – F 5′-CCAATAGACACCCCTTTTGAACC-3′, R 5′-TTTTCCTCCCACTTGCTCACC-3′; contig 16260 – F 5′-CACTGAAGAGGGAAACAAGCAAAG-3′, R 5′-AAGGTGGAATGTGAGCGTGATG-3′; contig 26389 – F 5′-CGATGAAACCAAAGCCGCTC-3′, R 5′-CCTCACCGACGCCTAAAATAGTG-3′; contig 30350 – F 5′-AGAGAGGAAGTCCAGAGCGAATG-3′, R 5′-CAACGGCAACATCGGCTTTAC-3′.

RAD haplotype confirmation was carried out as above except that the forward primer was within the RAD site sequence. Four bi-allelic RAD site sequences and associated contigs were PCR amplified and the product was TOPO-TA (Invitrogen) cloned. Four to seven individual clones were successfully sequenced from each region using the forward and reverse primers (T3, T7) present in the cloning vector. Sequence text files of the forward and reverse reads were analyzed using MacVector (7.1.1, MacVector, Inc.). After stripping vector sequences the files were aligned along with the contig sequences from our assembly and compared visually. Three of the 4 regions sent for Sanger sequencing had the expected two different haplotype sequences, with more than one read sampling each haplotype. The 4^th^ primer pair confirmed the presence of the correct SNPs determined by the haplotype analysis, but gave ambiguous results, with more than 2 different haplotype sequences appearing, suggesting the region was either a repetitive sequence or that there were technical problems with the amplification of this region. Primer pairs: haplotype 2 – F 5′ CCTGCAGGAAAGGAGACCG 3′, R 5′ CATGTGTGAGTGCATGAGCTCG 3′; haplotype 3 – F 5′ CCTGCAGGAAGCCGTGC 3′, R 5′ CTAATCCATGAACATTTCCTCTGG 3′; haplotype 5 – F 5′ CCTGCAGGGACATCCACAGTC 3′, R 5′ CACAAGTCACCAATAAAACATGTGG 3′; haplotype 8 – F 5′ CCTGCAGGATTTTTGGAAGTGTTG 3′, R 5′ AGACACACAGAGCTGGATGCAGG 3′.

### Partial-digest RAD paired-end library construction for Illumina sequencing (stickleback fosmid, *E. coli*)

Multiple digestion reactions were set up for each DNA sample containing either 1.0 µg of each fosmid DNA sample (BP11.12H 7e2 sox9) or 2.0 µg of *E. coli* REL606 genomic DNA, 5.0 µl 10× Buffer 4, 100 µg/ml BSA and 2 U of *Nla*III or *Sau3A*I (NEB). The reactions were incubated at 37°C in a 50 µl reaction volume for multiple lengths of time in order to achieve a spectrum of partially-digested to fully-digested DNA fragments. Digested samples were heat-inactivated for 20 min at 65°C and run out on a 1.0% agarose gel. A smear of DNA approximately 1.0–5.0 kb was isolated for each sample with a clean razor blade and purified. The isolated samples were quantified and the remaining DNA was ligated to enzyme-specific P1 Adapters (1.0 µ*M*), modified Illumina© adapters (2006 Illumina, Inc., all rights reserved; *Nla*III-P1 top oligo: 5′-AATGATACGGCGACCACCGAGATCTACACTCTTTCCCTACACGACGCTCTTCCGATCTCATG-3′; *Nla*III-P1 bottom oligo: 5′-Phos-AGATCGGAAGAGCGTCGTGTAGGGAAAGAGTGTAGATCTCGGTGGTCGCCGTATCATT-3′; *Sau3A*I-P1 top oligo: 5′-AATGATACGGCGACCACCGAGATCTACACTCTTTCCCTACACGACGCTCTTCCGATCT-3′; *Sau3A*I-P1 bottom oligo: 5′-Phos-GATCAGATCGGAAGAGCGTCGTGTAGGGAAAGAGTGTAGATCTCGGTGGTCGCCGTATCATT-3′), as described above, at a 10∶1 molar ratio of adapter to DNA ends (assuming an average genomic DNA fragment length of 3.25 kb). Samples were heat-inactivated for 20 min at 65°C and randomly sheared to an average size of 500–800 bp. Sheared samples were purified, run out on a 1.0% gel and DNA smears 200–800 bp (200–1200 bp for the *E. coli* samples) were isolated and purified. DNA polishing and 3′ dA-overhang addition was carried out as described. PE-P2 ligations were carried out with 0.5 µl PE-P2 Adapter. Samples were purified, eluted in 50 µl and quantified. 20 ng of template was used in a 100 µl, 14-cycle Phusion PCR amplification with 25 µl Master Mix and 2.0 µl amplification primer mix. Libraries were cleaned and gel purified, excising DNA ∼250–850 bp (250–1250 bp for the *E. coli* samples) in a triangle shape as above, diluted to 10 n*M*, and sequenced on the Paired-end module of the Genome Analyzer II following Illumina protocols for 40×80 bp reads.

### Long-insert RAD paired-end library construction for Illumina sequencing (*E. coli*)

8.0 µg of *E. coli* ATCC 11303 genomic DNA was digested for 60 min at 37°C in a 100 µl reaction volume containing 10.0 µl 10× Buffer 4 and 80 units (U) *Sbf*I-HF (New England Biolabs [NEB]). Following heat-inactivation, *Sbf*I fragments were ligated to 1.0 µl of barcoded *Sbf*I-P1 Adapter (1 µ*M*), a modified Illumina© adapter (2006 Illumina, Inc., all rights reserved; top oligo: 5′-ACACTCTTTCCCTACACGACGCTCTTCCGATCTxxxxxTGC*A-3′ [barcode - CCATA]; bottom oligo: 5′-Phos-xxxxxAGATCGGAAGAGCGTCGTGTAGGGAAAGAGTG*T-3′), with 2.0 µl T4 DNA Ligase. Samples were again heat-inactivated for 20 min at 65°C. The sample was split in two and randomly sheared in the bioruptor for 2 and 5 sec on low, to an average size of 5.0 and 2.0 kb, respectively. The sheared samples were purified and half the sample was run out on a 0.8% agarose gel. A smear of DNA approximately 1.0–10 kb was isolated with a clean razor blade and purified. DNA polishing was carried out in a 100 µl volume reaction with 2.0 µl Blunt Enzyme Mix. 3′ dA-overhang addition was performed as before using 15 U Klenow Fragment. PE-P2 ligation was carried out with 1.0 µl PE-P2 Adapter. The sample was purified, eluted in 50 µl and quantified. The resulting long-insert RAD template that was ∼1.0–6.0 kb or greater in size was processed in two ways: 1) 80 ng long-insert RAD template was amplified in a 200 µl 18-cycle Phusion PCR reaction with 100 µl Master Mix and 8.0 µl modified Illumina© amplification primers (10 µ*M*, 2006 Illumina, Inc., all rights reserved; Phospho-long-P1-forward primer: 5′-Phos-AATGATACGGCGACCACCGAGATCTACACTCTTTCCCTACACGACGCTCTTCCGATC*T-3′, Phospho-P2-reverse primer: 5′-Phos-CAAGCAGAAGACGGCATACG*A-3′) and a 5 min extension for amplification of longer fragments; and 2) 400 ng long-insert RAD template was run on a 0.8% agarose gel, ∼2.0 kb and larger was excised in a triangle shape and purified. 40 ng of the purified template was used in a 100 µl 18-cycle Phusion PCR amplification with the same primers. Both libraries were run out on 0.8% agarose gels, amplified products were excised in a triangular fashion and purified. Amplified samples were used as template (400 ng for library 1, 200 ng library 2) in circularization reactions and incubated overnight at 30°C in 200 µl or 100 µl reactions, respectively, containing 10 µl 10× T4 DNA Ligase Buffer (NEB) and 6.7 µl (∼20 U) T3 Ligase (Enzymatics) per 100 µl reaction volume. Samples were treated with 1.0 µl Plasmid-Safe (Epicentre) per 100 µl reaction volume for 20 min at 37°C, then the enzyme was heat-inacitvated for 30 min at 70°C. 4.0 µl 0.5 *M* EDTA (pH 8.0) per 100 µl reaction volume was added to each sample and the remaining circular DNA was randomly sheared to an average size of ∼600 bp. The DNA was purified, its ends were polished once more and then re-circularized overnight in a 200 µl reaction volume with 20 µl 10× T4 DNA Ligase Buffer and 6.7 µl T3 Ligase. Following Plasmid-Safe treatment with 2.0 µl of enzyme, the reaction was purified with a MinElute Spin column and eluted in 13 µl. 2.0 ng of the eluate was used as template in a 50 µl, 18-cycle Phusion PCR amplification with 25 µl Master Mix and 2.0 µl modified Illumina© amplification primers (10 µ*M*, 2006 Illumina, Inc., all rights reserved; long-P1-forward primer: 5′- AATGATACGGCGACCACCGAGATCTACACTCTTTCCCTACACGA**C**GCTCTTCCGATC*T -3′, long-P2-reverse primer: 5′-CAAGCAGAAGACGGCATACGAGATCGGTCTCGGCATTCCTGCTGAAC**C**GCTCTTCCGATC*T-3′). Libraries were gel purified, excising DNA ∼450–750 bp, diluted to 10 n*M*, and sequenced on the Paired-end module of the Genome Analyzer II following Illumina protocols for 40×80 bp reads. With a combined read length of 120 bp and DNA fragment sizes averaging 600 bp, no more than 25% of reads should contain the junction between sheared ends of the long RAD circular molecules.

## Supporting Information

File S1 **Stickleback contigs for SNP calling part 1.** This text file lists in fasta format the assembled contigs used for aligning the raw reads for SNP calling. The fasta header is in Velvet format. The file was split into pieces in order to upload online.
(TXT)

File S2 **Stickleback contigs for SNP calling part 2.** This text file lists in fasta format the assembled contigs used for aligning the raw reads for SNP calling. The fasta header is in Velvet format. The file was split into pieces in order to upload online.
(TXT)

File S3 **Stickleback contigs for SNP calling part 3.** This text file lists in fasta format the assembled contigs used for aligning the raw reads for SNP calling. The fasta header is in Velvet format. The file was split into pieces in order to upload online.
(TXT)

File S4 **Stickleback SNPs.** This text file lists the polymorphisms called between the stickleback samples, using the S1–S3 contig files as the reference. Explanation of output: 32831_TGCAGGAGTATTGACTGAACTTTTAACCCCCATGCTGCT_NODE_1_length_338_cov_9.647929; 212 C low-110 [C/A] high-141 C; Each SNP is described by two lines of text. The first line gives the contig and should match a header in the S1–S3 files. The second line gives the position of the SNP in the contig, the nucleotide of the SNP, and then the genotype of the two samples. In the above case, low-110 is heterozygous for the SNP and high-141 is homozygous. 32831_TGCAGGAGTATTGACTGAACTTTTAACCCCCATGCTGCT_NODE_1_length_338_cov_9.647929; 34 G low-110 [G/T] high-141 - Here low-110 is heterozygous for the SNP, and high-141 genotype was not called, usually because of low coverage at that position. 32831_TGCAGGAGTATTGACTGAACTTTTAACCCCCATGCTGCT_NODE_1_length_338_cov_9.647929; 76 T low-110 [T/A] high-141 [T/A]; Here both samples are heterozygous for the SNP.
(TXT)

File S5 **Stickleback fosmid contigs.** This text file lists in fasta format the assembled contigs from the partial-digest RAD-PE prep of stickleback fosmids.
(TXT)

File S6 ***E. coli*** **full genome contigs part 1.** This text file lists in fasta format the assembled contigs from the partial-digest RAD-PE prep of *E. coli* REL606 strain. The file was split into pieces in order to upload online.
(TXT)

File S7 ***E. coli*** **full genome contigs part 2.** This text file lists in fasta format the assembled contigs from the partial-digest RAD-PE prep of *E. coli* REL606 strain. The file was split into pieces in order to upload online.
(TXT)

File S8 ***E. coli*** **full genome contigs part 3.** This text file lists in fasta format the assembled contigs from the partial-digest RAD-PE prep of *E. coli* REL606 strain. The file was split into pieces in order to upload online.
(TXT)

File S9 ***E. coli*** **full genome contigs part 4.** This text file lists in fasta format the assembled contigs from the partial-digest RAD-PE prep of *E. coli* REL606 strain. The file was split into pieces in order to upload online.
(TXT)

File S10 ***E. coli*** **Long-Insert RAD-PE contigs.** This text file lists in fasta format the assembled contigs from the long-insert partial-digest RAD-PE prep of *E. coli*, unknown strain.
(TXT)

File S11 **Stickleback SNP RAD-PE.** This text file contains the perl scripts used to assemble contigs from the stickleback RAD-PE library sequence reads to create Files S1 & S4.
(PL) Click here for additional data file.

File S12 **Stickleback fosmid RAD-PE.** This text file contains the perl scripts used to assemble contigs from the stickleback fosmid partial-digest RAD-PE library sequence reads and create File S5.
(PL) Click here for additional data file.

File S13 ***E. coli*** **RAD-PE.** This text file contains the perl scripts used to assemble contigs from the *E. coli* partial-digest RAD-PE library sequence reads and create Files S6, S7, S8, S9.
(PL) Click here for additional data file.

File S14 ***E. coli*** **Long-Insert RAD-PE.** This text file contains the perl scripts used to assemble contigs from the *E. coli* long-insert RAD-PE library sequence reads and create File S10.
(PL) Click here for additional data file.
